# Palliative Care for Children with Central Nervous System Malignancies

**DOI:** 10.3390/bioengineering5040085

**Published:** 2018-10-13

**Authors:** Peter H. Baenziger, Karen Moody

**Affiliations:** 1 Peyton Manning Children’s Hospital, Ascension St. Vincent, 2001 West 86th Street, Indianapolis, IN 46260, USA; peter.baenziger@ascension.org; 2 MD Anderson Cancer Center, University of Texas, 1515 Holcomb Blvd., Unit 87, Houston, TX 77030, USA

**Keywords:** palliative care, child, brain, neoplasm, neuropathic pain, pain, symptoms, hospice

## Abstract

Children with central nervous system (CNS) malignancies often suffer from high symptom burden and risk of death. Pediatric palliative care is a medical specialty, provided by an interdisciplinary team, which focuses on enhancing quality of life and minimizing suffering for children with life-threatening or life-limiting disease, and their families. Primary palliative care skills, which include basic symptom management, facilitation of goals-of-care discussions, and transition to hospice, can and should be developed by all providers of neuro-oncology care. This chapter will review the fundamentals of providing primary pediatric palliative care.

## 1. Introduction

Neurological tumors comprise the second most common malignancy of children and adolescents, with an incidence of approximately 5000 children affected per year [1]. In addition, neurological tumors represent the most common cause of cancer-related death in children and adolescents [2]. Neurological tumors generally fall into high or low grade strata and generally higher grade tumors have poorer survival, however, patients with either high grade or low grade tumors suffer the adverse effects of chemotherapy, radiation, surgery, and direct disease sequelae, which may be ameliorated with palliative care.

Palliative care for children is critical to providing optimal care for some of the most vulnerable patients [3,4]. Providers caring for children with life-threatening illness should have a fundamental understanding of how to assist patients and families in establishing goals-of-care and essential pain and symptom management skills, the mainstays of the field of hospice and palliative medicine [5,6,7]. The American Academy of Pediatrics outlines principles to guide palliative care practice, including: (1) Providers have an obligation to ensure interventions are only used when potential benefits outweigh risks; (2) the goal of palliative care is to enhance quality of life despite the disease trajectory; (3) palliative care focuses on symptoms and conditions; and (4) palliative care teams work towards healthy bereavement for the family of the patient. Palliative care is generally provided by multidisciplinary teams to address the wide variety of burdens faced by patients suffering from serious illness, including neurologic tumors [8,9,10]. There is consensus that the ideal team includes a physician, nurse, social worker, spiritual advisor, and a child life therapist [5].

## 2. Facilitating Discussion and Decisions

A critical aspect of primary palliative care is to assist with establishing goals-of-care with patients and families. Establishing goals first requires some determination of the patient’s and family’s values (herein, simply “the family”) and priorities; this is achieved across a series of conversations. Examples of common values and priorities include: Staying out of the hospital, controlling pain, spending time with loved ones, and achieving milestones, such as graduation [11,12]. These values are discussed in the context of the patient’s illness, treatment options, and prognosis to establish priorities and goals of care and to facilitate medical decision-making. The medical decisions faced by parents for children with brain tumors are often numerous, frequent, high-stakes decisions and should align with these established goals of care. A key to facilitating these difficult conversations is to communicate in a way that “meets people where they are”—essentially, by not giving too much or too little information (which can be assessed through direct inquiry) and by enabling the patient’s and family’s values to drive the conversation. Goal setting is best accomplished when there is a consideration of the broader view of a patient and family, beyond the disease context, to include their personal lives, their community, and their social, emotional, and spiritual wellness [13].

Communicating well is important to patients, decreases uncertainty, and may improve hope and reduce decision regret [14]. Most parents would prefer to receive information about palliative care treatment options for their children than for this information to be withheld [14]. Young adults with cancer who have experienced an honest prognostic discussion with their providers demonstrate increased trust in providers, increased peace and hope, and decreased distress [15]. See Table 1 for communication tools [16].

Palliative care principles obligate providers and families to provide a developmentally appropriate explanation of disease and the burdens expected to the patient within the context of their values, culture, and preferences [5]. Earlier discussion of prognosis may be beneficial to allow for processing of information and allowing for earlier integration of prognosis into decision making [10]. Intentional, compassionate discussions of death should include consideration of the child’s developmental maturity, understanding of death, prior experiences with death, and the cultural and religious milieu in which the child lives, including family preference about if, when, and how information is disclosed to the patient [5]. Families should be encouraged to discuss the child’s fears, meeting them at a developmentally appropriate level [20,21]. It is important to note that studies demonstrate children are often aware of their grave prognosis before parents or care teams engage in a formal conversation and that children may avoid the topic to protect their parents [22].

A common decision put to families is whether and when to enroll in hospice. Hospice is a comprehensive home care program, with 24-hour, on-call nurse visits, home medication delivery, continuous 1:1 nursing if needed, and bereavement support. Eligibility for hospice only requires a reasonable possibility of death within six months; a do not resuscitation order is not required. Hospice utilizes a multidisciplinary approach focusing on comfort and quality of life. Advanced care planning is important for end-of-life care, including the patient’s/family’s preference for location of death. Unique to pediatrics is the concept of “concurrent care”, which allows children to “concurrently” receive both curative therapies and hospice [23].

Another decision many families face is Phase I trial enrollment, which may provide families with a sense of hope, legacy, and dignity, despite a minimal likelihood of objective disease response. The burdens of the trial should be balanced with the family’s sense of benefit. Theoretically, enrollment on a clinical trial near end of life can complicate comfort-directed care plans because of study-related requirements, however, available data suggest that patients that enroll on Phase I trials have similar timing and frequencies of “do not resuscitate” (DNR) discussions, hospice enrollment, and established DNR orders to those that do not enroll on such trials [24].

Since medical decisions in the palliative setting are value-based, conflicts may arise between family decision makers and medical teams regarding the goals of care and specifics of treatment. Conflict can be addressed at both a provider level and an institutional level. Providers of palliative care can mitigate disagreement by “shifting” to a more curious stance, in which “one focus[es] on learning more about the perspectives of both parties, exploring what may be a complex web of actions that contributed to the conflict.” [25]. A simple example of one of the ways one can make such a shift is repeating back what one hears a family member express as their hope; “so I hear you saying you hope there is a surgery that will stop your son’s cancer”. Identifying a common ground among identified hopes of family members is a helpful first step in negotiating a resolution. Many other practical communication tools have been summarized and are worthy of further reading and practice [26]. At an institution level, it is important to have a process of conflict resolution [27]. Such policies usually include purposeful communication efforts, opportunities for families to seek second opinions, and consultation with an ethics committee.

### Symptom Burden

The symptoms experienced by children with brain tumors are varied and can be quite burdensome [8]. The location of the tumor may predict likely burdens: Supratentorial tumors are associated with seizures, coma, and nausea and vomiting; infratentorial tumors are associated with ataxia; brain stem tumors are associated with speech disturbances, cranial nerve paralysis (swallowing dysfunction), and tetraparesis [28,29]. In addition to the most common symptoms of other childhood cancers (fatigue, pain, dyspnea), children with brain tumors can also suffer dysphagia and dysarthria, hearing and vision loss, paralysis, seizures, agitation, headaches, and cognitive and behavioral changes [30,31,32]. 

## 3. Oromotor Dysfunction and Secretions

Expert experience provides the bulk of treatment recommendations for oromotor dysfunction, which include speech therapy evaluation, thickening of feeds, and treatment with steroids to acutely reduce edema.

Secretions often become difficult for patients to manage as they lose their oral motor proficiency and their alert mental status. The goal of treatment is to decrease respiratory distress, aesthetic distress of secretions, and to attain maximum patient comfort. Medicines used are muscarinic anticholinergics, such as glycopyrrolate, scopolamine, or atropine. Glycopyrrolate does not cross the blood-brain barrier and thus spares patients from central nervous system side effects. Scopolamine patches pose a convenient transdermal delivery system and are approved for patients greater than 45 kg. Atropine ophthalmic 1% solution can be given sublingually; 1 drop every 4 h providing 0.5 mg atropine per dose [33]. Other treatment strategies are regular oral care and suction, lateral positioning of the patient, and music to dampen the noise. Families can be reassured that the rattling noise is not painful (an analogy to snoring works well) and deep suction will generally cause more harm than benefit.

### Nutrition and Hydration

As oromotor dysfunction progresses to dysphagia, nutrition and hydration often become concerns that need particular attention due to the fundamental nature of care through feeding. Nutrition and hydration should be framed by prognosis, risks (such as aspiration pneumonia), benefits (child’s enjoyment of favorite foods, tastes, and experiences; social interactions surrounding meals), and focused on the experience of the patient. For those patients who can no longer feed by mouth yet express hunger or other symptoms due to a lack of intake, the benefits to intervening may outweigh the risks. Generally, if a child is likely to live longer than 90 days, it is reasonable to use more invasive solutions (surgically placed feeding tube, total parenteral nutrition) if such procedures are aligned with family and patient values. If the prognosis is shorter, a nasogastric or nasojejunal feeding tube is more appropriate, with decreased risks, and foregoing feeds and fluids altogether is also an acceptable option for some. The burdens of feeds should not be forgotten; patients may feel discomfort from the feed itself, the tubes, or may have adverse responses to their change in appearance. When the primary goal is patient comfort, even when oral feeding poses an aspiration risk, it is reasonable to allow small amounts of intake for enjoyment. Conversely, when the primary goal is patient comfort, it is ethically sound to forego nutrition and hydration that are burdensome to the patient. Finally, offering hydration without nutrition or in the setting of impending death, often leads to increased symptom burden (dyspnea, swelling, pain) compared to mild dehydration [34].

## 4. Communication Difficulties

Children with neurologic tumors may suffer frustrating decreases in their ability to communicate due to dysarthria, aphasia, or hearing deficits [35,36]. Assisted communication devices require significant time to learn, thus they are less useful than simple tools, like dry erase boards or symbol books [37,38,39]. Speech, occupational, and physical therapies can have a significant impact by slowing the decline of key functional quality of life skills, such as speech and swallowing [8,40].

## 5. Headache

Headache occurs in around 36% of patients with brain tumors [41]. The primary treatment modality is pharmacologic, including acetaminophen, opioids, and dexamethasone. Medications that address neuropathic pain can be beneficial, but often take a week or more to be effective. Low dose methadone (0.04 mg/kg/dose BID) is an opioid that also has *N*-Methyl-d-aspartic acid (NMDA) receptor antagonist properties and can be particularly efficacious in cancer related headache, but may necessitate consultation with a palliative care or pain specialist comfortable with dosing and monitoring [42]. Methadone can be compounded into a 10 mg per mL solution for easy sublingual administration near end of life. Morphine can be similarly compounded into highly concentrated sublingual solutions. When using steroids for headache, the lowest possible effective dose should be used, and weaning should be considered after control of symptoms. NSAIDs (non-steroidal anti-inflammatory drugs) are typically avoided due to risk of bleeding at the tumor site and due to the risk of gastric ulcers with concurrent use of steroids.

## 6. Seizures

Seizures occur in 30–50% of patients with brain tumors. Seizures cause distress to the patient and family and may also cause worsening cognitive and behavioral functioning [43,44]. The treatment goals are to control seizures and to optimize periods of alertness. There is no evidence to support routine prophylactic antiepileptic use in the absence of a documented seizure history and these agents may cause sedation or agitation [45]. For acute seizure treatment, the benzodiazepines are most helpful. Diazepam and lorazepam can be given rectally, and lorazepam and midazolam are both absorbed intranasally [45].

For chronic seizure suppression, levetiracetam, as a broad acting staple, has few adverse effects, is well tolerated, and is often effective [46]. Many patients experience a brief, initial period of aggression on levetiracetam; if it does not resolve within several weeks, vitamin B6 supplementation has been shown in a small study to reduce the behavior change [47]. Pediatric neurology consultation is prudent for refractory seizures, status epilepticus, or patient intolerance due to adverse effects.

Because neurologic tumors are associated with dysphagia, concentrated sublingual compounds and rectally administrated antiepileptic medications often become necessary. Pharmacists are an important resource in this regard. Diazepam is the primary rectal choice as it comes in a suppository and acts more quickly than other antiepileptics given by this route. 

## 7. Nausea and Vomiting

Nausea and vomiting in the setting of pediatric neural tumors may be due to treatment or direct effects of the tumor. The vomit center receives input from two central nervous system controlling areas (chemoreceptor trigger zone; nucleus tractus solitarius) [48] and one peripheral input source (vagus nerve) [49], which use a multitude of neurotransmitters (histamine, acetylcholine, dopamine, serotonin, neurokinin) [50]. Treatment goals are to reduce nausea and vomiting to the point that patients can interact with family, can take by mouth foods they enjoy, and maintain nutrition and hydration.

Consider whether nausea is due to direct pressure from tumor, chemotherapy, toxins (uremia, hepatic failure, hypercalcemia), vestibular pathology, or gastrointestinal pathology (obstruction, ulcers, mucositis, constipation) [48]. When the cause is increased intracranial pressure, steroids are a first-line treatment. When chemotherapy or radiation-induced nausea and vomiting is expected, prophylaxis is recommended, and several published guidelines for children exist [51,52]. For the best control of nausea and vomiting, one should utilize both a scheduled medication as well as an as-needed medication; choosing medications from different classes is also ideal. See Table 2 for a list of antiemetics and dosages. Non-medical therapies can include hypnosis [53,54] and acupuncture [55]. Additionally, practical remedies, such as treating constipation, removing noxious smells, opioid rotation, emotional support [56], and modifying meals to be smaller and more frequent, can be helpful [55].

## 8. Pain

Pain is reported by many children and adolescents with brain tumors, and may be due to the cancer itself, the treatments received, and the procedures performed [33]. Most patients with neurological tumors benefit from pain medicines at some point in their disease process (84%) [57]. Neuropathic pain caused by the tumor can be particularly challenging to treat and may require polypharmacy for best results.

Other manifestations of “total pain” [58] are addressed hereafter, including psychological, social, emotional, and spiritual elements [59]. These must be treated in parallel to physical pain as they are codependent.

Treatment of pain in the patient with neuro-oncological disease begins with the foundations of pain in other clinical care arenas: Assessment, identifying type of pain, intervention, and reassessment. Validated scales should be used whenever possible to track progress. The FLACC scale [60] is useful for caregiver observation of infants and other non-verbal patients. For children 3 years and older, the Wong Baker FACES scale gives an age appropriate visual scale and, for ages 9 years and older [61], a simple 0–10 numerical rating scale is appropriate.

The two fundamental types of pain are nociceptive and neuropathic. Nociceptive pain includes somatic (muscle and bone) or visceral (organs) pain. Somatic pain is typically localized and described as “sharp”, “aching”, or “throbbing” whereas visceral nociceptive pain is poorly localized and described in a variety of ways (“gnawing”, “cramping”, “pressure”). Neuropathic pain is typically due to damage to nerves and is described as “burning”, “needles”, or “numbness”, or is aggravated by touch [62]. Much of the pain experienced in neuro-oncology patients is of mixed type as brain tumors directly press on neuronal tissue and therefore a mixed approach is commonly undertaken.

Nonpharmacologic treatments should accompany the pharmacological approach for patients with any severity of pain; these include environmental changes, treating comorbid symptoms [63] (fear, anxiety, depression), and integrative approaches [64], such as: hypnosis [65], mind-body therapies [66,67], heat and cold stimulation, massage, acupuncture, physical therapy, exercise, biofeedback, art therapy, guided imagery, and distraction.

Pharmacologically, mild nociceptive pain is treated with acetaminophen; moderate to severe nociceptive pain is treated with opioids. For those with pain at least every other day, a long-acting, scheduled agent as well as a short acting as-needed agent should be employed. Short acting agents should be 10–20% of the daily opioid morphine equivalent dose and given every 2–4 h as needed. Methadone is a very useful long acting *mu*-agonist and NMDA antagonist [68] agent available in a variety of forms for easy pediatric dosing. Consultation with a provider comfortable with dosing and management may be necessary [69,70]. Fentanyl patches provide transdermally delivered opioid that can be used in opioid tolerant patients; however, doses are fixed and therefore can be difficult to titrate in smaller patients. In addition, morphine, and oxycodone are available in extended release formulations for use in patients as an oral alternative to methadone and fentanyl patches. These agents come in uncrushable, fixed-dose tablets, which precludes their use in small patients and those that cannot swallow pills. Dosing of extended release (ER) morphine and oxycodone is generally based on a patient’s current daily opioid use. For example, a patient taking morphine 5 mg every 4 h around the clock is taking 30 mg per day. This patient would be started on 15 mg morphine ER twice daily. Breakthrough pain doses of 10–20% of daily doses can be offered in addition every 4 h; in this case, 3–6 mg would be appropriate. See Table 3 for nociceptive pain medications and dosages.

In patients with recalcitrant pain, a pain titration may become necessary. The precept of a pain titration is to quickly titrate medication to relief, or dose-limiting side effects. A pain titration is accomplished with interval intravenous (IV) dosing of morphine (or hydromorphone), beginning with an appropriate initial dose for weight and re-assessing response every 30 min. After each assessment using a validated pain assessment scale, additional morphine doses are given as follows: (1) If no relief and pain is >7, administer and increase dose by 50–100%, (2) if partial relief, but pain still >5, give one-half to 1 times the initial dose, and (3) if pain score is 4 or less, then no dose is given at the 30-min assessment. This process is repeated until the patient’s pain is 4 or less, or intolerable side effects occur. The total milligrams of opioid required to achieve pain control is calculated by dividing the total morphine dose given by the total number of elapsed hours since initiation of the pain titration, thus providing the hourly morphine rate. With this information, one can calculate an equivalent daily methadone dose, fentanyl patch, or a basal morphine IV/SQ infusion rate. Breakthrough pain treatment with additional doses of morphine are then added at 20% of the daily morphine dose and offered every 3 h IV prn. Alternatively, a patient-controlled analgesia (PCA) pump can be started with a morphine basal rate equivalent to the calculated hourly morphine rate, an added “PCA” or “demand” dose of 100% of the hourly rate (range 50–200%) and a timed “lock-out” of 10 min. Re-assessment of pain should occur multiple times per day; increases in pain and opioid use should be followed by dose adjustments. In the terminal setting, it is not uncommon for 80% of the daily opioid used each day to be delivered continuously via methadone or an IV opioid infusion, and for patients to need a twice daily upward titration [71]. Continuous opioid rate should be adjusted as often as every 6–8 h (time to reach steady state) to attain the goal of demand dosing being required less than twice per hour while awake, and to allow for uninterrupted periods of sleep. Demand and interval breakthrough pain doses can be adjusted after three doses. Additional bolus doses of two times the PCA demand dose can be given every 1–2 h while awaiting therapeutic opioid levels. See Table 4 for initial pediatric PCA dosing.

Advantages of PCA use include: (1) The ability of patients to titrate pain medication to relief; (2) reduced delay in addressing changes in pain intensity; and (3) gives children and young adults some control over their symptom management. Contraindications to the use of PCA are all relative in the end-of-life setting. However, PCA is not an ideal delivery mechanism for patients that are delirious, encephalopathic, or physically unable to press the demand button. If the patient is actively dying, and goals of care are well established, and comfort-directed, then PCA by-proxy (nurse or family member) is appropriate and an often-optimal way to provide pain management [72]. 

Common opioid side effects include pruritis, nausea, sedation, and constipation. In general, sedation is dose-related and should be avoided, except at end-of-life if consistent with goals. Constipation should be anticipated with all opioids except methadone, and prophylaxis is appropriate in the setting of “around the clock” opioid treatment. Orders for antihistamine are also indicated for as needed use for pruritis. Nausea can be treated with antiemetics. In general, when side effects are not tolerated, opioids can be rotated as patients respond differently to different opioids and, in general, hydromorphone is less likely to cause pruritis than morphine. When rotating opioids, the equivalent opioid dose should be calculated and then reduced by 25% to account for incomplete cross-tolerance. See Table 5 for side effects and suggested treatments.

Reassessment is critical to pain control and should be performed at a frequency consistent with the medications used (consider expected time-to-onset and duration of action) and pain severity. Children suffering pain deserve aggressive pain control, which may require escalating doses of opioids significantly and rapidly. There is no empiric upper limit dose on opioids; however, an individual may reach their upper limit when he/she experiences increased side effects without additional benefit to pain control [73]. The treatments should follow the “2-step ladder” determined by the World Health Organization: Step 1 includes acetaminophen and/or NSAIDs for mild pain +/− non-opioid adjuvants; and Step 2 adds opioids for more severe pain [74]. Medication choices should be personalized for the patient, considering allergies, comorbidities, drug interactions, adverse effects, social support, and cultural perspectives.

Neuropathic pain in children is treated primarily with gabapentinoids, and/or tricyclic antidepressants, which decrease central nervous system (CNS) excitatory neurotransmission [75]. Again, methadone, with its NMDA antagonism, is also a helpful medication when pain is severe and chronic [48]. Clonidine, topiramate, and duloxetine may also be useful, particularly when agitation, headache, or depressed mood are present, respectively. See Table 6 for neuropathic agents. Adjuvant medications to treat comorbid painful conditions, such as muscle spasms, abdominal cramps, and chemotherapy induced peripheral neuropathy, are shown in Table 7.

Though beyond the scope of this text, pain management can also include palliative radiation and surgical approaches, such as dorsal rhizotomy. Consultation with radiation oncology, neurosurgery, pain medicine, and/or hospice and palliative medicine specialists is prudent in patients with recalcitrant pain. In the rare case where pain control cannot be achieved, the goals-of-care may be consistent with palliative sedation, which necessitates consultation with palliative care specialists [71,76,77,78].

## 9. Altered Mood

As brain tumors progress, they often (in 60% of children) cause mood changes, such as depression or anxiety, which are important [79], yet under-addressed, forms of suffering. In the setting of life-limiting illness, such mood disorders are likely biochemical in nature as well as “supratentorial” or cognitive in nature. Therefore, appropriate care assesses and treats both etiologies. Adolescents, in particular, demonstrate accelerated transitions, notably an “illness transition” in which one identifies with the illness as being part of their being, and a “developmental transition” in which development behaviors change in response to the disease [80]. However, their growth comes with feelings of sadness, anxiety, difficulty speaking with their parents, and fear of being alone; and unlike pain, which is addressed more than 80% of the time, these emotional symptoms are far less frequently addressed (around 45% of the time) [81]. Depressed mood or anxiety should be treated based on severity at presentation. For all patients with mood changes and unmanaged stress (e.g., fear, changes in function, uncontrolled symptoms), integrative approaches and non-pharmacological options including cognitive behavioral therapy, family therapy, and massage/relaxation therapy can offer benefit. Music, along with other forms of art, are increasingly used to help patients and family members express their emotions [82,83,84]. For patients presenting with mild symptoms rooted primarily in an uncertainty of the future and fear of death, dying support through attention, education, and expressive supportive counseling is appropriate. Therapeutic counseling is provided by a variety of clinicians, using a myriad of techniques, including: Meeting the patients where they are in development, allowing time and presence to validate emotions, and compassionate, honest discussion of the anticipated disease trajectory. For example, family pastors, hospital chaplains, and bedside nurses (among others) can assess the child’s belief system and address spiritual/existential fears, as well as engage in reflective listening, prayer, touch, silence, and ritual [85,86,87]. Social workers play a critical role in helping patients achieve their personal goals of legacy and in leveraging the patient’s support network for direct support to the patient [88,89]. Psychologists can assess the patient for specific fears and provide cognitive behavioral counseling [90].

For moderate symptom burden, cognitive behavior therapy in addition to medications, such as selective serotonin reuptake inhibitors (SSRIs), selective norepinephrine reuptake inhibitors (SNRIs), and tricyclic antidepressants, should be initiated and selected primarily based on the secondary beneficial effects (i.e., SNRIs for neuropathic pain component, SSRIs for weight gain, etc.). For severe mood symptoms, medications and supportive therapies should be initiated together, psychology and/or psychiatry consultation should be obtained, and disposition should be considered (i.e., admission to facility for safety if suicidal).

Children and adolescents laden with symptoms and the weight of disease, may express their burden through requests for a hastening of death. American Academy of Pediatrics (AAP) guidelines do not support physician-assisted-death; the request should be met with normalization, compassionate care, and an aggressive focus on relieving burdens and increasing quality of life [91,92,93]. Psychological consultation is important. It is important to remember and communicate that foregoing interventions and thus allowing a disease to progress along its natural course to death is ethically sound, is not suicide, and is not physician-assisted death [94,95] Elucidating the patient’s voice about their hopes and fears is powerful; Voicing My Choices [96] and Five Wishes [97] are two studied, standardized tools [98].

## 10. Agitation, and Altered Mental Status

Many patients with brain tumors demonstrate agitation, or delirium [35,41,47].

Agitation can be caused by direct tumor effects, medical complications (e.g., uremia), and medications (e.g., opioid). Benzodiazepines (midazolam, lorazepam, diazepam, clonazepam) at the lowest possible dose are the primary treatment, especially if anxiety is present, followed by neuroleptics (haloperidol, thioridazine, chlorpromazine, risperidone), and alpha-agonists (clonidine) [49]. Neuroleptics are particularly helpful when delirium is present. See Table 8 for dosing recommendations.

An altered mental status is the most common symptom as children with tumors of the brain approach death (75% of children within the last week of life; 85% overall) [33,47]. This usually manifests as a slow decrease in consciousness over days to weeks. While treatment options are few at this stage of disease, it is important that clinicians provide anticipatory guidance to family and other providers.

## 11. Care of the Family, Caregiver, and Siblings

Palliative care teams maintain the goal of caring not only for the patient, but also for the entire family as they grieve and bereave [99]. The complicated emotion and process of grief and bereavement, respectively, begin at the time of diagnosis and accelerate as attention is turned from cure of disease to palliation of symptoms. Finding meaning and realistic hoped-for goals can soothe patients and families in this phase of care. 

While oncology, palliative care, and other interdisciplinary teams have matured areas of disease and symptom management, families report significant deficiencies in psychological care near the end of life [100]. Many family members report unaddressed psychospiritual distress and significant caregiver burdens [93]. Consultation with physical therapy, occupational therapists, child life specialists, art therapists, chaplains, and hospices can provide additional supportive resources. Siblings deserve particular attention and developmentally appropriate interventions, such as cognitive behavioral therapy, art, and play therapies, and close bereavement follow-up.

Similarly, regardless of the prognosis or the choices in care that parents select, disease-specific or pediatric oncological support communities can provide a community, validation, and education to the entire family unit; providers should try to connect families to such a group whenever possible.

## 12. Summary

Pediatric patients suffering from CNS tumors face difficult clinical decisions and carry a significant symptom burden. Given the high risk of mortality, communication of prognosis and goal-setting can be challenging. Supporting caregiver decision making requires honest, empathic communication and attention to the family values and emotions. Ongoing symptom assessment and management is a must to optimize quality of life and reduce suffering. Providers caring for these children should have a basic knowledge of palliative communication skills and pain and symptom management, with access to specialist palliative care providers as needed.

## Figures and Tables

**Table 1:** Palliative care communication tools.

Delivering Bad News	Verbal Responses to Emotion	Non-Verbal Response to Emotion
“SPIKES”: [17] -Setting: Prepare the setting for the conversation and minimize distractions. -Perception: Assess the caregiver’s perception of the clinical information. -Invitation: Ask permission to deliver new information. -Knowledge: Provide the main message up front, simply. -Emotion: Respond empathically to emotion. -Strategy/Summary: Summarize the encounter and what will happen next.	“NURSES”: [18,19] -Naming the emotion statement: “I hear frustration in your voice.” -Understanding statement: “I understand this is upsetting news.” -Respect statement: “I can see how dedicated you have been to your son’s care over these three months.” -Support statement: “We are here to help you and your family.” -Explore statement: “Tell me about what you were hoping to hear today.” -Silence: Providing silence in the room can passively, yet explicitly recognize emotions.	“SOLAR”: [20] -Squarely face the patient. -Open body posture. -Lean towards the patient. -Eye contact. -Relaxed body posture.

**Table 2:** Antiemetics.

Class	Drug	Dose	Forms	Notes
NK-1 Antagonist	Aprepitant (Emend)	Day 1: 3 mg/kg PO (max 125 mg) Day 2, 3: 2 mg/kg PO (max 80 mg)	Capsule, suspension	Approved for chemotherapy induced nausea/vomiting (CINV). Assess for CYP3A4 & 2C9 drug interactions Minimal data exists on the use of fosaprepitant in children <12 years
Steroid	Dexamethasone (Decadron)	10 mg/m^2^ IV/PO daily (reduce to 5 mg/m^2^ if using with aprepitant)	IV, tablet, solution	This is the CINV dose; alternate dosing is used for brain edema
5HT3 Antagonist	Ondansetron (Zofran)	0.15 mg/kg/dose IV/PO q8 hours (max 8 mg/dose)	IV, tablet, oral disintegrating tablet, solution	5HT3 antagonists have equivalent efficacy at comparable doses
	Granisetron (Kytril)	0.04 mg/kg IV daily or PO q12 hours (max 1 mg/dose) age >12 years: 1–2 mg PO/IV q12 hours	IV, tablet, solution (custom compounded), patch (available as outpatient prescription for adolescents)	
	Palonosetron (Aloxi)	0.02 mg/kg IV once prior to chemo. If necessary, may re-dose 72 hours later	IV	
Phenothiazine	Promethazine (Phenergan)	0.25 mg/kg PO/IV q6 hours (max 25 mg/dose)	IV, tablet, syrup, suppository, topical gel	Contraindicated in children <2 years old. Anticholinergic.
	Prochlorperazine (Compazine)	0.1 mg/kg/dose IV/PO q6 hours (max 10 mg/dose)	IV, tablet, suppository	Contraindicated in children <2 years old or <9 kg; anticholinergic and anti-dopaminergic; risk of extrapyramidal symptoms
Prokinetic	Metoclopramide (Reglan)	0.1–0.5 mg/kg IV/PO q6 hours (max 10 mg) Adolescents: 5–10 mg IV/PO q6 hours	IV, tablet, suspension	Risk of tardive dyskinesia, especially with prolonged use; may use with oral diphenhydramine. Anti-dopaminergic
Benzodiazepine	Lorazepam (Ativan)	0.04 mg/kg IV/PO q8 hours (max 2 mg/dose)	IV, tablet, suspension	Risk of sedation, respiratory depression, coma, and death when used with opioids
Atypical Antipsychotic	Olanzapine (Zyprexa)	0.14 mg/kg/dose PO qHS (max 5–10 mg/dose)	tablet, orally disintegrating tablet	Antidopaminergic, anticholinergic, and 5HT2 antagonist.
Cannabinoid	Dronabinol (Marinol)	5 mg/m^2^ PO BID-QID (max 10 mg/dose)	Capsule	Contraindicated with sesame oil hypersensitivity
Antihistamine	Diphenhydramine (Benadryl)	0.5 mg/kg PO q6 hours	Oral, elixir	Avoid IV use due to dependency and sedation risk. Also, may use to manage EPS side effects.
Anticholinergic	Scopolamine (Transderm Scop)	1.5 mg patch changed q72 hours	Patch	For use in patients >45 kg
Butyrophenone	Haloperidol (Haldol)	3–12 years old start 0.05 mg/kg/day divided BID-TID >12 start 0.5 mg per dose BID-TID, up to 4 mg/dose q 6 hours	PO tabs/IV/SC	Anti-dopaminergic. Risk of severe extrapyramidal symptoms, prolonged QT and granulocytopenia

**Table 3:** Nociceptive agents.

Drug	Route	Dose						Notes
Acetaminophen	Oral, IV, Rectal	10 mg/kg IV q6 hours or 15 mg/kg PO q4 hours.						Avoid in liver disease or consult with hepatologist / GI specialist regarding dosing.
	Initial short-acting dose in an opioid naïve patient							
	**Route**	**Dose**	**Onset (min)**	**Peak Effect (h)**	**Duration (h)**	**Initial Scheduled Dosing in Opioid Naïve Patients**	**Available Oral Dose Formulations**	
Tramadol	PO	1–2 mg/kg/dose (max initial dose 25–50 mg); Maximum daily dose 400 mg	30–60	1.5	3–7	Short-acting: Every 4–6 hours. Long acting: Every 12 hours	Short-acting: 50 mg tablets Long-acting: 100, 200, 300 mg tablets	Not approved for children less than 18 years of age. May lower seizure threshold. Increased risk of Serotonin Syndrome.
Hydrocodone	PO	0.1–0.2 mg/kg/dose (max 5–10 mg)	10–20	1–3	4–8	Short-acting: Every 6 hours	Short-acting in combination with acetaminophen: 5, 7.5, 10 mg tablets; 2.5 mg/5 mL liquid	Hydrocodone used for pain is only available in combination with acetaminophen or ibuprofen.
Morphine	PO	0.2–0.5 mg/kg/dose (max 5–15 mg)	30	0.5–1	3–6	Short-acting: PO: Every 4 hours. Long-acting: Every 12 hours	Short-acting: 15, 30 mg tablets; 10 mg/5 mL, 20 mg/5 mL, 20 mg/1 mL liquid. Long acting: 15, 30, 60, 100, 200 mg tablets	Short-acting preparation can be compounded into very concentrate SL drops (20 mg/mL). Long acting morphine for opioid tolerant patients only.
	IV/SC	0.05–1 mg/kg/dose (max 2–3 mg)	5–10	N/A	N/A	Every 4 hours	N/A	
Oxycodone	PO	0.1–0.2 mg/kg/dose (max 5–10 mg)	10–15	0.5–1	3–6	Short-acting: Every 4 hours. Long-acting: Every 12 hours	Short-acting: 5, 15, 30 mg tablets; 5 mg/5 mL, 20 mg/mL liquid. Long-acting: 10, 15, 20, 30, 40, 60, 80 mg tablets	Available alone or in combination with acetaminophen. Long acting form for opioid tolerant patients only.
Hydromorphone	PO	0.03–0.06 mg/kg/dose (max 1–3 mg)	15–30	0.5–1	3–5	Short-acting: Every 4 hours; long acting: Once daily	Short-acting: 2, 4, 8 mg tablets; 1 mg/mL liquid Long-acting: 8, 12, 16, 32 mg tablets	Long acting form is for opioid tolerant patients only.
	IV/SC	0.01–0.015 mg/kg/dose (max 0.5–1.5 mg)	15–30	N/A	4–5	Every 4 hours	N/A	
Methadone	PO/SC/PO	0.04 mg/kg/dose BID and titrated weekly to effect	30 min (PO)	3–5 days	Increases with repeater doses up to 60 hours		Tablet, Liquid	Consult expert provider. May prolong QTc; check baseline ECG.

**Table 4:** Intravenous patient controlled analgesic starting dose recommendations.

Drug	Demand Dose	Lockout Interval	Continuous Rate	4 h Limit
Morphine	0.025 mg/kg (max 2 mg)	10–12 min	0.015 mg/kg/h	0.3–0.4 mg/kg
Hydromorphone	0.005 mg/kg (max 0.3 mg)	6–10 min	0.003 mg/kg/h	0.06–0.08 mg/kg
Fentanyl	0.25 mCg/kg (max 20 mCg)	6–10 min	0.015 mCg/kg/h	3–4 mCg/kg

**Table 5:** Common or important opioid adverse effects and treatments.

Adverse Effect	Treatments
Constipation	-Polyethylene glycol: 0.5–1.5 GM/kg PO daily. -Senna: 4.3–17.2 mg/day PO (2–6 years), 6–50 mg/day PO (6–12 years), 12–100 mg/day (12 years and older).
Pruritis	-Hydroxyzine (preferred for least sedation): 50 mg/day PO divided every 6 h (<6 years), 50–100 mg/day PO divided every 6 h (≥6 years). -Diphenhydramine: 6.25 mg q4 hours as needed (2–6 years), 12.5–25 mg PO q4 hours as needed (6–12 years), 25–50 mg PO q 4 hours as needed (12 years and older). -Opioid rotation (switch opioids with 25% dose reduction). -Naloxone 0.25–1 microgram/kg/hour IV infusion.
Urinary Retention	-Oxybutinin: 0.2 mg/kg/dose (max 5 mg/dose) PO TID (≤5 years old), 5 mg/dose TID for >5 years old. -Relieve with catheterization, then lower dose or rotate opioid with 25% dose reduction.
Euphoria/Dysphoria	-Lower dose or rotate opioid with 25% dose reduction.
Somnolence	-Lower dose or rotate opioid with 25% dose reduction. -methylphenidate 0.3 mg/kg/dose, (max initial dose 2.5–5 mg/dose) given before breakfast and before lunch (≥6 years old)

**Table 6:** Neuropathic agents.

Drug	Dose	Notes
Gabapentin	Day 1: 5 mg/kg/dose (max 300 mg/dose) PO at bedtime. Day 2: 5 mg/kg/dose (max 300 mg/dose) PO BID. Day 3: 5 mg/kg/dose (max 300 mg/dose) PO TID. Dose may be further titrated to a maximum dose of 50 mg/day (and generally no more than 1800 mg/day).	Comes in a liquid. May cause drowsiness, dizziness, and peripheral edema. Dose adjust for renal impairment.
Pregabalin	75 mg BID.	Initial adult dose; can titrate up to 300 BID max.
Clonidine	Oral: Immediate release: Initial: 2 mCg/kg/dose every 4 to 6 h; increase incrementally over several days; range: 2 to 4 mCg/kg/dose every 4 to 6 h. Topical: Transdermal patch: May be switched to the transdermal delivery system after oral therapy is titrated to an optimal and stable dose; a transdermal dose is approximately equivalent to ½ to 1 × the total oral daily dose.	Limited data available for pain in children and adolescents. Helps with opioid withdrawal, helps with sleep. Can lower BP. Good for dysautonomia pain.
Topiramate	-6–12 years (weight greater than or equal to 20 kg): 15 mg PO daily for 7 days, then 15 mg PO BID. -≥12 years: 25 mg PO at bedtime for 7 days, then 25 mg PO BID and titrate up to 50 mg PO BID. -Maximum daily dose 200 mg.	May cause acidosis, drowsiness, dizziness, and nausea. Dose adjust for renal impairment and hepatic dysfunction.
Amitriptyline	-0.1 mg/kg PO at bedtime. -titrate as tolerated over 3 weeks to 0.5–2 mg/kg at bedtime. -Maximum: 25 mg/dose.	Consider for continuous and shooting neuropathic pain. Caution use in patients with arrhythmias. May cause sedation, arrhythmias, dry mouth, orthostasis, and urinary retention. Caution use in patients with seizures; avoid MAOIs, other SSRIs, or SNRIs due to potential for serotonin syndrome.
Duloxetine	Approved for anxiety in children >7 years. Start with 30 mg capsule at bedtime and can titrate up to 60 mg qHS.	Antidepressants can increase suicidal thinking in pediatric patients with major depressive disorder. Duloxetine may increase the risk of bleeding events. Concomitant use of aspirin, nonsteroidal anti-inflammatory drugs, warfarin, and other anti-coagulants may add to this risk. Taper slowly.

**Table 7:** Adjuvant pain treatments.

Drug	Indication	Dose	Notes
Dexamethasone	Inflammation, Nerve compression	-1 mg/kg/day IV or PO in divided doses every 6 h). -Maximum: 16 mg/day. Use lowest effective dose.	May cause impaired healing, infection, thrush, hyperglycemia, weight gain, myopathy, stomach upset, psychosis, emotional instability.
Diazepam	Muscle spasms	Oral: Children: 0.12 to 0.8 mg/kg/day in divided doses every 6 to 8 h.	
Tizanidine	Muscle spasms	Children 2 to <10 years: Oral: 1 mg at bedtime, titrate as needed. Children ≥10 years and Adolescents: Oral: 2 mg at bedtime, titrate as needed.	Oral: Titrate initial dose upward to reported effective range of: 0.3 to 0.5 mg/kg/day in 3 to 4 divided doses; maximum daily dose: 24 mg/day.
Cyclobenzaprine	Muscle spasms	Greater than or equal to 15 years old: 5 mg PO three times daily Maximum 30 mg/day.	
Dicyclomine	Abdominal cramping	Infants ≥6 months and Children <2 years: Oral: 5 to 10 mg 3 to 4 times daily administered 15 min before feeding. Children ≥2 years Oral: 10 mg 3 to 4 times daily Adolescents: Oral: 10 to 20 mg 3 to 4 times daily. If efficacy not achieved in 2 weeks, therapy should be discontinued.	
5% lidocaine patch	Nociceptive or neuropathic pain	1–3 patches applied daily (depending on size) up to 12 h per day.	Can be cut to fit.
OTC creams	Nociceptive or neuropathic pain	Apply topically to localized areas of neuropathic pain BID-TID.	
Prescription creams: Diclofenac cream; compounded neuropathic agents	Nociceptive or neuropathic pain	Apply topically to localized areas of neuropathic pain BID-TID.	
Ice, heat	Nociceptive or neuropathic pain		

**Table 8:** Medical treatments for agitation.

Drug	Route	Dose	Available Oral Dose Formulations
Lorazepam	PO, IV	0.05 mg/kg/dose PO/IV q4 hours; max single dose 2 mg.	Tablet: 0.5, 1, 2 mg. Oral solution 2 mg/mL.
Diazepam	PO, IV, IM	0.12–0.8 mg/kg/day PO divided q6 hours. 0.04–0.2 mg/kg IV/IM q2 hours, max. 0.6 mg/8 h.	Tablet: 2, 5, 10 mg. Solution: 1 mg/mL, 5 mg/mL.
Clonazepam >6 years	PO, IV	<30 kg: 0.01–0.03 mg/kg/day PO divided q8 hours; increase by 0.25–0.5 mg/day q3 days; maximum 0.1–0.2 mg/kg/day PO divided q8 hours. >30 kg: 1.5 mg/day PO divided q8 hours; increase by 0.5–1 mg q3 days; maximum 20 mg/day.	Tablet
Midazolam	PO, IV, intranasal	500–750 mCg/kg PO once prior to procedure.	Oral solution: 2 mg/mL.
Haloperidol ≥3 years	PO, IM	Oral: 0.01 mg/kg/dose 3 times daily as needed. Starting dose max 0.5 mg/day. Titrate slowly as directed.	Tablet: 0.5, 1, 2, 5, 10, 20 mg. Oral solution: 2 mg/mL.
Chlorpromazine ≥6 months	PO, IV	Initial: 0.55 mg/kg/dose PO every 4–6 h as needed. Common initial dose 10–25 mg. Max daily dose ≤5 years old: 50 mg/day; >5 years old 200 mg/day.	Tablet: 10, 25, 50, 100 mg.
Risperidone	PO	>5 years old and 15–20 kg: 0.25 mg/day qHS; >20 kg 0.5 mg/day qHS or divided BID. Can titrate up 100% after 4 days.	Tablet (and orally dissolving tablet): 0.25, 0.5, 1, 2, 3, 4 mg. Oral solution: 1 mg/mL.
Clonidine	PO, IV, transdermal	0.1–0.5 mg PO q8 hours; titrate up slowly every 3 days; wean upon discontinuing.	Tablet: 0.1, 0.2, 0.3 mg. Extended release: 0.1 mg. Patch: 0.1, 0.2, 0.3 mg/day.
